# Therapeutic Avenues to Modulate B-Cell Function in Patients With Cardiovascular Disease

**DOI:** 10.1161/ATVBAHA.124.319844

**Published:** 2024-05-30

**Authors:** James W. O’Brien, Ayden Case, Claudia Kemper, Tian X. Zhao, Ziad Mallat

**Affiliations:** 1 Division of Cardiorespiratory Medicine, Department of Medicine, Victor Phillip Dahdaleh Heart and Lung Research Institute, University of Cambridge, United Kingdom (J.W.O., A.C., T.X.Z., Z.M.).; 2 Complement and Inflammation Research Section, National Heart, Lung, and Blood Institute, National Institutes of Health, Bethesda, MD (C.K.).; 3 Department of Cardiology, Royal Papworth Hospital, Cambridge, United Kingdom (T.X.Z.).; 4 Unversité de Paris, Inserm U970, Paris Cardiovascular Research Centre, France (Z.M.).

**Keywords:** atherosclerosis, cardiovascular diseases, cell survival, immunization, T-lymphocytes

## Abstract

The adaptive immune system plays an important role in the development and progression of atherosclerotic cardiovascular disease. B cells can have both proatherogenic and atheroprotective roles, making treatments aimed at modulating B cells important therapeutic targets. The innate-like B-cell response is generally considered atheroprotective, while the adaptive response is associated with mixed consequences for atherosclerosis. Additionally, interactions of B cells with components of the adaptive and innate immune system, including T cells and complement, also represent key points for therapeutic regulation. In this review, we discuss therapeutic approaches based on B-cell depletion, modulation of B-cell survival, manipulation of both the antibody-dependent and antibody-independent B-cell response, and emerging immunization techniques.

The adaptive immune system plays a crucial role in the induction and progression of atherosclerosis. Several B-lymphocyte subsets are involved in the disease process, playing distinct roles (Figure 1). Innate B1 cells are atheroprotective through their production of natural IgM antibodies, which bind and neutralize the proinflammatory effects of oxidation-specific epitopes (OSEs). In contrast, mature adaptive B2 cells have been shown to be proatherogenic through their role in germinal center formation and the production of class-switched IgG and IgE antibodies and through their role in supporting proatherogenic dendritic cell maturation and T-cell activation.^1–3^ Monoclonal antibody (mAb)–mediated B2-cell depletion reduces atherosclerotic lesion development in several mouse models without affecting plasma cholesterol levels.^4^ More recently, there has been a focus on the role of marginal zone B cells (MZBs), which are innate-like B2 cells, with evidence suggesting that they play atheroprotective roles both through the production of anti-OSE IgM antibodies and through the modulation of the T follicular helper response.^5–7^ Furthermore, selective subsets of B cells are specialized in the production of specific cytokines, through which they affect the development and progression of atherosclerosis. In particular, innate response activator B cells promote atherosclerosis through the production of GM-CSF (granulocyte-macrophage colony-stimulating factor) and regulatory B cells (Bregs), which may be atheroprotective through the production of IL (interleukin)-10. Interestingly, a B2 lineage-derived memory B-cell subset characterized by the production of GM-CSF has been shown to regulate IL-10 production by B cells in other autoimmune diseases.^8^

**Please see www.ahajournals.org/atvb/atvb-focus for all articles published in this series.**

**Figure 1.**
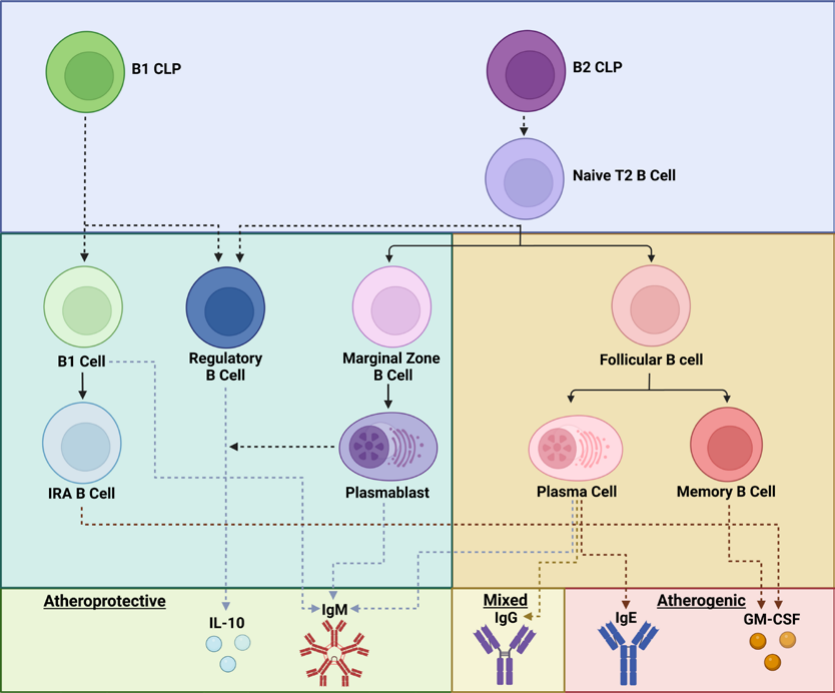
**B-cell subset overview.** Overview of B-cell developmental subsets and their generalized impact on atherosclerosis.^106–108^ CLP indicates common lymphoid progenitor; GM-CSF, granulocyte-macrophage colony-stimulating factor; IL, interleukin; and IRA, innate response activator.

B cells have also been implicated in the progression of atherosclerosis post-myocardial infarction (MI).^9^ Disease progression was associated with increased accumulation of IgG antibodies in atherosclerotic lesions, driven by germinal center activation, which could be attenuated following B cell depletion with a CD (cluster of differentiation)-20 monoclonal antibody.^9^

The function of B cells in cardiac remodeling post-acute MI has also been studied in preclinical models: following acute MI, B lymphocytes are activated by danger-associated molecular patterns and orchestrate the mobilization and recruitment of Ly6C^hi^ monocytes into the infarcted area in a CCL7 (chemokine CC motif ligand 7)-dependent manner.^10,11^ Treatment with anti-CD20 mAb depletes mature B2 cells, reduces CCL7 production, and limits cardiac Ly6C^hi^ monocyte infiltration, leading to significant improvement in the recovery of heart function.^10^ Recent work has identified MZB cells as the specific B2-cell subset responsible for detrimental post-MI remodeling.^12^

Here, we discuss several ways to modulate the B-cell response in cardiovascular disease (CVD), ranging from B-cell depletion, attenuating B-cell survival, and altering antibody production or cell-cell interactions.

## B-CELL DEPLETION

The use of B cell–depleting agents is not a novel idea and has been used since 1997, with rituximab being first licensed for the treatment of lymphoma.^13^ Since then, there have been numerous agents licensed for the treatment of hematological malignancies and neurological and rheumatological conditions.^14^

**Figure 2.**
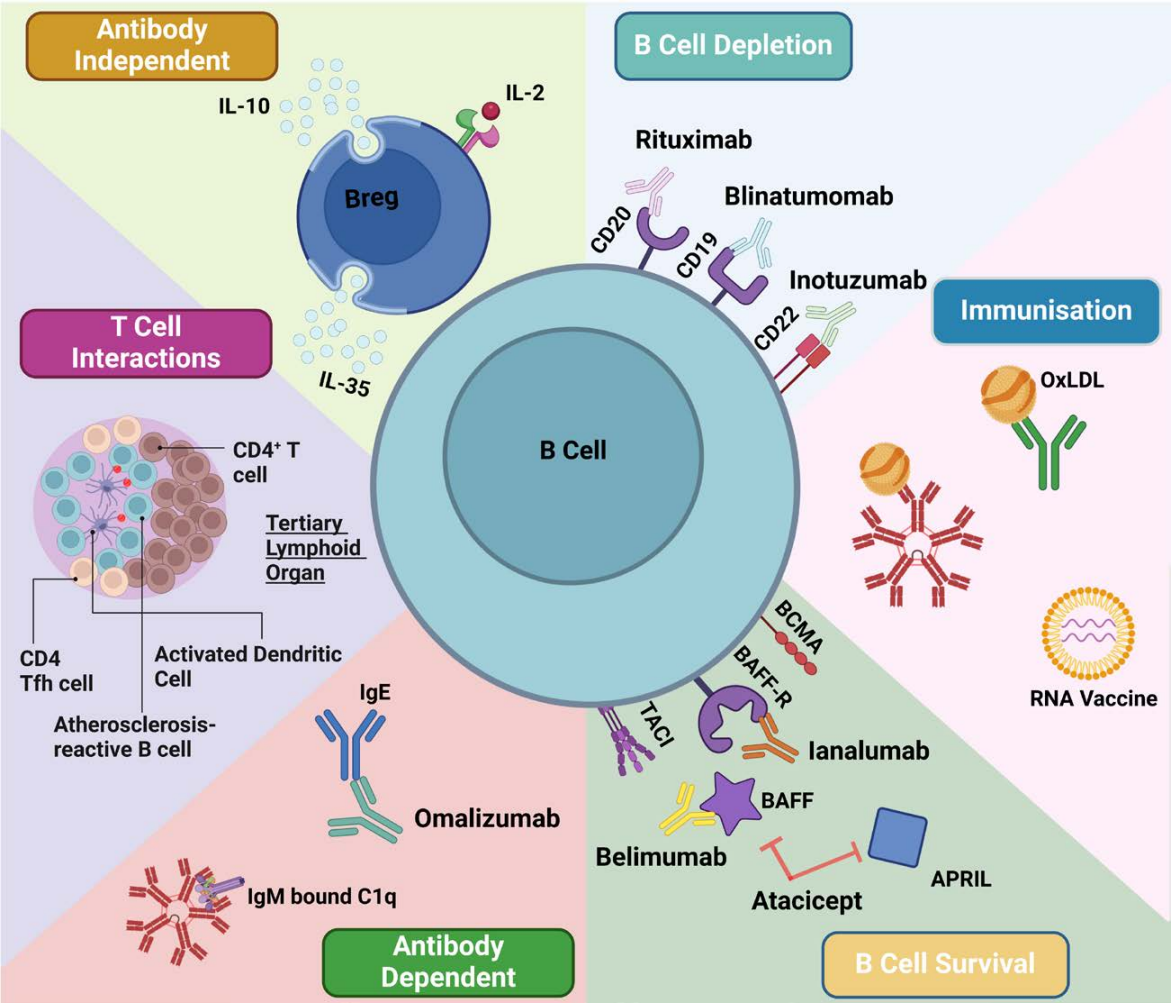
**Avenues for B-cell modulation in cardiovascular disease.** B cells have multiple therapeutic targets aimed at modulating their function; shown are B cell–related therapeutic targets and currently available drugs. APRIL indicates a proliferation-inducing ligand; BAFF, B-cell activating factor; BAFF-R, B-cell activating factor receptor; BCMA, B-cell maturation antigen; Breg, regulatory B cell; CD, cluster of differentiation; IL, interleukin; OxLDL, oxidized low-density lipoprotein; TACI, transmembrane activator and calcium-modulator and cyclophilin ligand interactor; and Tfh, T follicular helper cell.

Preclinical studies support that B-cell depletion using an anti-CD20 mAb post-MI results in reduced infarction size and improved left ventricular (LV) contractility.^10^ These effects are accompanied by a reduction in atherosclerotic lesion development.^4,15^ Building on this preclinical data, RITA-MI (Rituximab in Patients With ST-Elevation Myocardial Infarction)^16^ was the first translational study (phase I/IIa) to test this hypothesis in humans. RITA-MI tested 4 doses of rituximab (200, 500, 700, and 1000 mg), starting the intravenous infusion within 48 hours of symptom onset, with safety as the primary end point (Figure 2). The trial confirmed that rituximab is safe to give in the post-MI period; with 24 patients receiving rituximab, 7 serious adverse events were reported; all were assessed to not be associated with the drug. Secondary study end points included changes in circulating B cells and their subsets. There was a significant depletion in circulating B lymphocytes within 30 minutes of treatment with all doses tested (mean reduction, 96.3% from baseline), indicating rituximab’s suitability for rapid B-cell depletion post-MI. The nadir of B-lymphocyte numbers was at day 6 post-infusion, and there was a dose-dependent repopulation at 6 months, without an accompanying effect on immunoglobulin levels.^16^

Building on the success of RITA-MI, a double-blinded, placebo-controlled, phase IIb clinical trial is now underway (RITA-MI-2; https://www.clinicaltrials.gov; unique identifier: NCT05211401) using LV function, assessed via cardiac magnetic resonance imaging at 6 months, to evaluate whether depletion of circulating B lymphocytes post–ST-segment–elevation MI has a measurable clinical effect in humans. In RITA-MI-2, 2 doses (200 and 1000 mg) of rituximab are being assessed. These 2 doses were selected based on the results of RITA-MI, which showed that the 2 doses result in similar acute B-cell depletion but differ in the timing of B-cell reconstitution. At 6 months, there was reconstitution of B-cell numbers in the 200-mg group compared with baseline, with proportionally more transitional B cells, which are known to have a regulatory phenotype.^17^ At the highest dose (1000 mg), there were still significantly depleted B-cell numbers at 6 months, although there remained a higher proportion of transitional B cells compared with baseline. RITA-MI 2 will also allow further assessment of the impact of transient (200 mg) versus longer term (1000 mg) B-cell depletion on atherosclerosis. These results will be followed-up with an ancillary study using ^18^F-FDG-PET/computed tomography at 6 months to assess the impact of rituximab on vascular inflammation post-MI.

There is further evidence for the potential benefit of rituximab in CVD. Reduced cardiovascular events were noted in patients who received rituximab following a kidney transplant during a follow-up period of 8 years compared with matched controls.^18^ The drug has also been explored as a therapy for multiple sclerosis on account of its ability to deplete proinflammatory B-cell subsets.^8^ Rituximab has further been proposed as a potential therapy in inflammatory dilated cardiomyopathy. Tschöpe et al^19^ presented a case series in which patients with biopsy-proven CD20^+^ B lymphocyte–associated inflammatory dilated cardiomyopathy, whose LV function had failed to respond to conventional medical therapy, had an improvement in LV function following treatment with rituximab. The full mechanism of this improvement is yet to be elucidated but is proposed to be related to a reduction in antimyocardial antibody production and B cell–mediated T-cell activation.

Targeting CD20, and, therefore, mature B2 cells, including MZB cells and potentially some B1-cell subsets in humans raises questions regarding the divergent roles of these B-cell subsets post-MI. Although the murine data suggest that selectively targeting MZB cells post-MI may offer a beneficial therapeutic target, long-term suppression may not be suitable and may be associated with adverse outcomes. MZB cells play a role in limiting the development of atherosclerosis through interactions with T follicular helper cells^6^ and through their production of atheroprotective anti-OSE IgM antibodies.^5,7^ Mice with a selective deletion of MZB cells have accelerated the development of atherosclerotic lesions.^5^ This suggests that transient inhibition/depletion of MZB cells is required post-MI and highlights the importance of assessing atherosclerosis progression in patients treated with B2 cell–depleting therapies. There are currently no dedicated therapies to transiently deplete MZB cells in humans. However, considering the results of RITA-MI, which showed transient depletion of B-cell subsets in the 200-mg rituximab dose group, we await the results of RITA-MI 2, which is powered to assess the impact of the 200-mg dose’s transient depletion on heart function and vascular inflammation in the post-MI period (NCT05211401).

There are other lymphocyte-depleting agents used in humans outside of CVD: blinatumomab (Figure 2) targets CD19, which is expressed on both plasma cells and B1 subsets^20^; both are known to be atheroprotective.^1,3^ Therefore, targeting CD19 is unlikely to be beneficial in atherosclerosis. Inotuzumab, which targets CD22, a protein long considered an inhibitory receptor^21^ on B cells, is linked to MZB cell development, with data showing that CD22 deficiency in mice results in reduced MZB numbers.^22^ Therefore, CD22 neutralization is unlikely to be beneficial in atherosclerotic CVD. There are currently no published data in humans showing a therapeutic benefit associated with these treatments in CVD; in fact, there are data that support that these agents may have a cardiotoxic effect.^23^

## B-CELL SURVIVAL

Several key B-cell survival factors have been investigated for their therapeutic potential. Two such factors are BAFF (B-cell activating factor), which is also known as TNFSF13B (tumor necrosis factor ligand superfamily member 13B), and APRIL (a proliferation-inducing ligand). The binding of both BAFF and APRIL combined with the BCR (B-cell receptor) binding to its antigen induces a cascade of intracellular signaling resulting in increased B-cell survival.^24^ These ligands have different binding affinities to the receptors involved in this pathway: BAFF-R (BAFF receptor) has strong selective affinity for BAFF, and while BCMA (B-cell maturation antigen) binds both BAFF and APRIL, it has a higher affinity for APRIL.^25^ TACI (transmembrane activator and calcium-modulator and cyclophilin ligand interactor) binds both BAFF and APRIL equally.^25^

Preclinical studies suggest that there may be potential therapeutic benefit in targeting the BAFF pathway. *Ldlr* ^*−/−*^ and *Apoe^−/−^* mice with deletion of BAFF-R have a significant reduction in B2 cells without an impact on B1a-cell subsets and are protected from the development of atherosclerosis.^26,27^ To further highlight the therapeutic potential of targeting the BAFF pathway, the use of a BAFF-R mAb protected against atherosclerosis in *Apoe^−/−^* mice.^28^

Preclinical studies also support that the benefit of modulation of the BAFF pathway may extend to the post-MI setting. Mice with BAFF-R deletion display significantly better LV function post-MI compared with controls.^10^ This effect is, in part, mediated through reduced numbers of B2 cells and a reduction in B2 cell–derived CCL7, which limits Ly6C^hi^ monocyte infiltration in the ischemic heart and improves cardiac remodeling post-MI.^10^

Currently, there are several drugs used to target B-cell survival in humans, mainly in hematological malignancies: belimumab, a BAFF mAb, blisibimod, a selective antagonist of BAFF,^29^ ianalumab, a mAb to BAFF-R,^30^ and atacicept, a TACI recombinant fusion protein that binds both BAFF and APRIL^31^ (Figure 2). Although the medications outlined here have been evaluated in either phase II or III clinical trials, to date, there have been no reports on cardiovascular outcomes. Belimumab is the only medication currently in use in routine clinical practice; it was first approved for use in systemic lupus erythematosus^32^ and continues to be used today for this purpose. Recent pilot data presented at the European Respiratory Society Congress highlight the potential benefit of belimumab in CVD.^33^ The group shows that following treatment with belimumab, there is a reduction in circulating anti-GRP78 (glucose-regulated protein 78) levels, which are associated with severe chronic obstructive pulmonary disease and the development of atherosclerosis, with no increase in infections. These preliminary data suggest potential utility in the use of belimumab as a treatment for atherosclerosis, but there is a clear need for longer follow-up data to investigate whether this reduction in anti-GRP78 translates into a reduction in cardiovascular events.

However, the literature does not support that inhibition of B-cell survival through direct inhibition of BAFF is completely favorable. Blockade of BAFF in either *Ldlr^−/−^* or *Apoe^−/−^* mice resulted in increased size and complexity of atherosclerotic plaques compared with controls despite depletion of B2 cells.^34^ These data suggest that, despite the potential benefits of targeting the BAFF pathway via the BAFF-R, targeting BAFF itself is not optimal. BAFF is expressed on a variety of cells, including dendritic cells, monocytes, and macrophages.^24^ Therefore, the use BAFF-targeting antibodies may impact the potentially atheroprotective, B cell–independent effects of BAFF.

Furthermore, a clinical trial using atacicept^31^ was halted prematurely due to an unexpected decline in serum IgG levels and the occurrence of serious infections.^35^ Another clinical trial using atacicept was forced to stop recruitment in one arm due to the death of 2 patients.^36^ The benefits of atacicept are also impugned in preclinical studies, as mice treated with atacicept had significantly lower circulating levels of atheroprotective IgM antibodies^37^ with no impact on atherosclerosis lesion size.^38^

APRIL has been shown to be atheroprotective in murine models. Mechanistically, this has been linked to the discovery of an alternative form of APRIL, termed noncanonical APRIL, which binds to HSPG (heparin-sulphate proteoglycan) 2, restricting the retention of LDL (low-density lipoprotein) in arterial walls and reducing macrophage retention, as well as the development of necrotic cores in atherosclerotic lesions.^38^ Serum levels of this noncanonical APRIL are inversely linked to cardiovascular and all-cause mortality, independent from other traditional risk factors.^38^ In a preclinical model of atherosclerosis, the use of an mAb, which specifically promotes APRIL binding to HSPGs, resulted in a reduction in atherosclerosis^38^; this offers a promising potential new therapeutic target.

## ANTIBODY-DEPENDENT FUNCTION

Several antibody-mediated B-cell functions have been implicated in the pathophysiology of atherosclerosis; some of these functions are atheroprotective, with others being atherogenic.

Several isotypes of antibodies, particularly IgM, are important initiators of the classical pathway of the complement cascade.^39^ Pentameric IgM, which is produced by B1 and MZB cells and found in the blood and lymph, initiates the complement cascade by binding antigen at multiple sites, inducing a conformational change that facilitates C1q binding. The subsequent cleavage of C3 (complement component 3) generates C3a and C3b, the latter of which is essential for efferocytosis of apoptotic cells and other immunogenic debris within atherosclerotic plaques. This complement-mediated removal of immune complexes is performed primarily by macrophages in a CR1 (complement receptor 1)-dependent manner; this function of complement facilitated by B1 and MZB cells is generally considered atheroprotective, contributing to plaque stability. In contrast to the generally positive effects of C3, the downstream, terminal activation of the complement cascade, particularly C5 (complement component 5) generation, is associated with plaque instability and acceleration of atherosclerosis.^39–41^ Liver-derived plasma circulating C5a levels control the activation state of the vascular endothelium through C5aR1 (complement C5a receptor 1)-mediated expression induction of adhesion molecules and foster immune cell diapedesis.^42^ However, C5 is also expressed by a range of immune cells, including monocytes and macrophages, and can control the activity of these cells in an intrinsic fashion. More specifically, engagement of a mitochondrial-expressed C5aR1, driven by intracellular C5a activation triggered upon cholesterol crystal sensing, induces IL-1β production in atherosclerotic plaque-resident human and mouse monocytes and macrophages.^43^ Pharmacological inhibition of mitochondrial-expressed C5aR1 in atherosclerotic plaque macrophages was associated with a reduction in IL-1β production and engagement of molecular pathways sustaining plaque stability.^43^ Interestingly, B cells also express cell-autonomous C5,^44^ and it may be worthwhile to investigate in the future whether the B cell–autonomous C5 system contributes to atherogenic B-cell responses.

Given the multifaceted roles of antibody-dependent complement activation in CVD, there is substantial interest in developing targeted therapeutics, with over 20 complement-specific drugs currently being investigated.^39,45^ Surprisingly, there has been limited success in bringing complement-targeting therapeutics to market for CVD patients, with Pexelizumab, an anti-C5 mAb, being a high-profile example.^46^ Recent insight into the extensive role of intracellular complement in immune modulation may provide a partial explanation for the attenuated impact of systemic, extracellular complement-targeting therapies.

In addition to the role of IgM in complement fixation and efferocytosis, the isotype is also a critical inhibitor of thrombosis and coagulation—the proximal drivers of MI and ischemic stroke secondary to atherosclerosis. Obermayer et al^47^ characterized the ability of IgM to bind procoagulatory microvesicles and directly inhibit their function. In a recent follow-up, the same group extends these findings, demonstrating that anti-OSE IgM inhibits thrombosis in a microvesicle-dependent manner by preventing the formation of neutrophil extracellular traps, a well-characterized contributor to thrombus formation.^48,49^ These studies establish coagulatory microvesicles as a potential therapeutic target in CVD.

Furthermore, IgM has emerged as a key regulator of autoreactive B-cell clones, mediating anergy to polymorphic self-antigens. Specifically, studies using transgenic mice have elucidated a tolerance mechanism by which endogenous antigen recognition induces IgM downregulation in self-reactive B cells, impinging on their ability to receive prosurvival signals and ultimately leading to their elimination.^50^

Despite the generally atheroprotective nature of IgM, other antibody isotypes have been shown to mediate more mixed effects, particularly IgG, which has generally been more closely associated with atherogenic effects than nonswitched antibody isotypes.^51^ Tay et al^52^ demonstrated that IgG-producing plasma cells mediate proatherogenic effects in a mixed chimeric mouse model, augmenting plaque inflammation and necrotic core size. These effects are thought to be the result of plaque-specific IgG antibody binding, with subsequent FcgR (Fc gamma receptor)-mediated natural killer cell activation and initiation of ADCC (antibody-dependent cellular cytotoxicity).^2,53^ The resulting effect on plaque stability is debated; with recent studies showing, counterintuitively, that IgG can augment both plaque size and stability.^54^

Other isotypes of immunoglobin, namely IgE, have also been implicated in atherosclerosis progression, with serum IgE levels being positively correlated with coronary artery disease severity.^55^ Previous studies have confirmed this finding, with serum levels of IgE, IgA, and IgG all shown to correlate with MI in men with increased CVD risk.^56^ The correlation of IgE with poor atherosclerosis outcomes is not surprising, as mast cells, through which IgE exerts its primary effector functions, have a well-characterized role in promoting vessel damage and generating inflammatory mediators.^57^ The correlation between IgE and atherosclerosis pathogenesis has been proven in IgE-deficient mice, which showed reduced susceptibility to diet-induced atherosclerosis.^58^ The role of IgE in atherosclerosis has also demonstrated utility as a therapeutic target, with anti-IgE mAbs eliciting atheroprotective effects in mice.^59^ Omalizumab, an mAb specific to the Fc portion of IgE, has been licensed for use in patients with severe asthma.^60^ However, an observational study assessing the long-term safety of omalizumab in patients with respiratory disease reported a higher incidence of serious adverse events in the omalizumab group compared with controls; importantly, there were differences in baseline characteristics, with the omalizumab group having double the number of patients with severe asthma, as well as a higher incidence of type 2 diabetes.^61^ These findings potentially complicate the development of similar IgE-based interventions in CVD.

Beyond the generalized role of various immunoglobulin isotypes in promoting efferocytosis or initiating an inflammatory cascade, secreted antibodies can mediate direct effects on target tissues with implications for CVD. A number of autoantibodies have been described in CVDs, spanning apolipoproteins, cell surface receptors, and myofibril proteins.^62,63^ Specific examples of self-antigen recognition directly mediating CVD include antibodies directed against adrenergic receptors and muscarinic receptors; these autoantibodies are implicated in the induction of atrial fibrillation.^64,65^ These findings have been extended in additional studies examining the relationship between various autoimmune diseases and new-onset atrial fibrillation, with a correlation being present across multiple conditions.^66^ These findings reveal an intriguing therapeutic target, with depletion of B cells producing antiadrenergic/muscarinic receptor antibodies being a potentially novel avenue for treating atrial fibrillation.

## ANTIBODY-INDEPENDENT B-CELL INTERACTIONS

B lymphocytes have other immune functions than their typically characterized role of antibody production, primarily mediated through cell-cell interactions. In particular, T-cell:B-cell interactions are critical regulators in atherosclerosis progression and resolution. T cell–facilitated germinal center reactions, the resulting somatic hypermutation/affinity maturation, and subsequent class switching of B cells can have both atherogenic and atheroprotective consequences. The outcome of such reactions depends on several factors, including the B-cell subset involved, the proximal immune microenvironment, and the particular antigen driving the reaction.

Aside from class switching from IgM, which has generally atheroprotective functions, to isotypes with more mixed effects (eg, IgG), the differentiation of B cells into various memory, effector, and regulatory subsets has important implications in atherosclerosis.

Over the last 2 decades, evidence has accumulated on the immunosuppressive role that the regulatory B lymphocytes (Bregs) play in autoimmune conditions in both mice and humans.^67,68^ Initially, Bregs were identified by their ability to secrete the immunosuppressive cytokine IL-10, but more recently, there is growing evidence of other cytokines, enzymes, and surface receptors produced or expressed by this heterogenous group of cells including IL-35, TGF-β (transforming growth factor-β), CD39, CD73, PD-L1 (programmed death-ligand 1), CD1d, and CD25.^69,70^

The most studied subset of Bregs remains the IL-10–producing subset.^70^ IL-10 mediates its suppressive effect via pathways involving T cells and macrophages.^71^ IL-10 inhibits inflammatory IFN-γ (interferon-γ) production by CD4^+^ T-cell subsets, inhibits antigen presentation by macrophages and monocytes, and reduces proinflammatory cytokine production by antigen-presenting cells.^72^ In addition to these generalized effects, IL-10 produced by Bregs, as other sources of IL-10, favors the formation of Tregs, which can then amplify the regulatory microenvironment, suppressing in particular proinflammatory T-cell subsets.^73–75^ The resulting tolerogenic environment has the potential to then lead to a systemic tolerance toward atherosclerosis-associated antigens, an idea explored in detail in several vaccine trials (see following section). The interaction between Tregs and B cells in atherosclerosis is likely an ongoing process, as Tregs may continue to interact with B cells in tertiary lymphoid organs (TLOs) after induction, potentially leading to the emergence of clones with enhanced specificity for disease-associated antigens.^76^

However, the role of Breg IL-10 in atherosclerosis is debated within the literature. Mice with a B cell–specific deficiency in IL-10 production had no significant difference in atherosclerotic lesions compared with controls, suggesting that B cell–derived IL-10 may not contribute an atheroprotective role. However, an adoptive transfer of Bregs from the lymph nodes of *Apoe^−/−^* mice led to attenuated neointimal formation in response to perivascular carotid artery injury,^77^ and this effect was lost with the use of an IL-10 neutralizing antibody or the transfer of B cells from IL-10–deficient mice. Further evidence has shown that transfer of IL-10^+^ B cells results in reduced infarction volume following middle cerebral artery occlusion in mice.^78^ In ischemic heart disease, adoptive transfer with IL-10^+^ B cells post-MI (induced) resulted in increased ejection fraction at 28 days compared with controls^79^; this effect was mediated through a reduction in Ly6C^hi^ monocyte infiltration and was negated when an IL-10 neutralizing antibody was administered.^79^ In aggregate, these data suggest that the endogenous production of IL-10 by B cells may not be sufficient to confer atheroprotection; however, if the production is supplemented or augmented, B cell–derived IL-10 may be beneficial. One putative therapeutic avenue for augmenting Breg IL-10 production is treatment with low-dose IL-2. Low-dose IL-2 has been shown to induce Tregs in humans, as seen in the LILACS trial (Low-Dose Interleukin-2 in Patients With Stable Ishcaemic Heart Disease and Acute Coronary Syndromes),^75^ with further analysis showing an increase in plasmablast populations enriched for a Breg gene signature.^73^ Furthermore, treatment of human peripheral blood and splenic B cells with IL-2 in vitro increases IL-10 secretion^73^ through the inhibition of BACH2 (BTB domain and CNC homolog 2), a transcriptional repressor that binds to the IL-10 promoter region, with further in vivo evidence of significant downregulation of BACH2 in patients treated with low-dose IL-2. Recently, a large phase II double-blinded placebo-controlled clinical trial investigating the impact of low-dose IL-2 on vascular inflammation post-MI (IVORY [Low-Dose Interleukin 2 for the Reduction of Vascular Inflammation in Acute Coronary Syndromes])^80^ concluded that a potentially important subanalysis is evaluation of whether IL-2–induced Bregs are associated with a reduction in vascular inflammation.

The induction of Tregs, whether via Bregs or exogenous mediators, has also become an important approach for addressing atherosclerosis pathogenesis. The ongoing ELLIPSE study (Effect of Low-Dose Interleukin-2 on the Immune Landscape of Human Atherosclerotic Plaques at Single Cell Resolution) intends to extend these findings by profiling immune microenvironment changes in the plaque tissue following low-dose IL-2 treatment (https://www.clinicaltrials.gov; unique identifier: NCT05975554).

Bregs are not only associated with IL-10 production; in recent years, there has been increasing interest in the role of IL-35 produced by Bregs,^81^ termed i35-Bregs, and the role it plays in CVD. These i35-Bregs have been shown to play an important role in immune regulation during autoimmune conditions^82^ by promoting atheroprotective Tregs.^83^ Interestingly, distinct Treg subsets characterized by IL-35 and IL-10 expression have been identified, a pattern mirrored in the emerging cytokine production profile of Bregs.^84^

## OTHER B-CELL INTERACTIONS WITH T CELLS

TLOs, lymphoid aggregates with lymph node–like attributes, have been identified within the artery wall in patients with atherosclerosis and may have an important role in local B-cell affinity maturation and isotype switching.^85^ Formation of TLOs is driven, in part, by B cell–derived lymphotoxin-αβ alongside other cytokines including IL-22.^86,87^ In atherosclerosis, the generation of these structures is suspected to result from the nonresolving inflammation characteristic of the disease, with TLO stability associated with T-cell homeostasis and deletion of TLOs being associated with aggravation of atherosclerosis in a mouse model.^86^ B cells play important roles in antigen presentation within these structures, and their subtypes and antigen specificity may determine whether TLO formation is atherogenic or atheroprotective.^86^ TLOs within the artery are also associated with the recruitment of B1 cells, specifically IL-10–producting B1b cells.^88^ The aggregate impact of these structures on atherosclerosis progression is still not fully understood.

## IMMUNIZATION AND RNA VACCINES

The primary B cell–oriented vaccine strategy for atherosclerosis is the induction of protective autoantibodies through immunization. In addition to lipid metabolism–targeting pathways, which can be examined in further detail here,^89–91^ several strategies targeting OSEs have been developed. These OSEs serve important immunologic roles as immunogenic markers for phagocytosis and inducers of sterile inflammation.^92^ Given their role in immune signaling, OSEs have been a significant focus for vaccine development.

One OSE that has shown potential as a vaccine target in atherosclerosis is malondialdehyde-modified LDL, a complex family of modified LDL molecules that are used as a biomarker for CVD.^93–95^ Indeed, a recent mouse study demonstrated that malondialdehyde-modified LDL vaccination is effective in generating a germinal center reaction and that mice lacking germinal center–derived plasma cells and antibodies have accelerated atherosclerosis progression.^96^ However, the GLACIER trial (Goal of Oxidized LDL and Activated Macrophage Inhibition by Exposure to a Recombinant Antibody), which examined the impact of supplementation with an IgG mAb targeting a malondialdehyde-modified epitope of ApoB-100 (orticumab) in stable atherosclerosis patients failed to reduce circulating or imaging markers of vascular inflammation.^97^ A phase II clinical trial assessing the impact of orticumab versus placebo in patients with psoriasis has been completed recently (https://www.clinicaltrials.gov; unique identifier: NCT04776629). The authors assessed arterial inflammation in the coronary arteries using the fat attenuation index score. There was a trend toward reduced coronary inflammation in response to orticumab. A subgroup analysis indicated that orticumab significantly reduced inflammation in the right and circumflex coronary arteries in patients with high inflammation at baseline. Similarly, orticumab significantly reduced the CaRi-Heart risk score (which integrates the fat attenuation index score with other clinical risk factors) in patients with high baseline inflammation. Although interesting, these results should be interpreted with caution given that the significant differences were seen only in unplanned post hoc subgroup analyses and that the placebo and orticumab groups differed with regard to baseline inflammation.^98^ Additional studies will be required to validate the results.

Another OSE-oriented approach utilizes molecular mimicry employed by pneumococci. Namely, antibodies against phosphorylcholine on the bacteria are cross-reactive with oxidized LDL epitopes, with immunization against the bacteria correlated with augmented anti-oxidized LDL IgM production.^99^ Following this, immunization of *Ldlr^−/−^* mice with streptococcus pneumoniae has been shown to limit atherosclerosis progression.^99^ This approach has been tested in humans in the AUSPICE trial (Australian Study for the Prevention Through Immunisation of Cardiovascular Events), where participants were given a pneumococcal polysaccharide vaccine and monitored for subsequent atherosclerosis markers.^100^ The investigators did not observe changes in atherosclerosis markers at a 2-year follow-up, and changes to long-term CVD risk are currently being assessed.^100^ The limited short-term effects of the intervention may be due to the relatively modest anti-oxidized LDL IgM antibody induction, the rapid waning of these atheroprotective antibodies, and the timing of the vaccine relative to the disease pathogenesis.^100^

Despite the conflicting preclinical and clinical data, it is clear that immunization against modified LDL epitopes remains an ongoing area of exploration for the generation of a B cell–mediated solution for preventing atherosclerosis. Given the recent success of mRNA-based vaccines for COVID-19 and the various ongoing trials for other diseases,^101^ it is conceivable that RNA vaccination using sequences coding for peptide mimotopes of OSEs or anti-OSE IgM antibodies could emerge as possible therapies.

A better characterization of B-cell subtypes and their antigen specificities will be required for the success of antigen-based and B cell–targeted immunotherapies. For instance, single-cell sequencing was used to identify and better characterize several subsets of B cells, including B1 cells,^102^ MZBs,^103^ or age-associated (ABC) B cells.^104,105^ Single B-cell RNA sequencing paired with BCR sequencing allows paired observations of B-cell subset phenotypes with their antigen specificities. The use of paired BCR sequencing can generate a better understanding of antigen-specific responses and guide therapeutic designs such as immunizations or the development of antibodies directed to specific BCR sequences.

## ARTICLE INFORMATION

### Sources of Funding

Work in the laboratory of Z. Mallat is also supported by Heart Research UK, a European Horizon 2020 grant (agreement: 899991; J.W. O’Brien), and the Leducq Foundation BCVD (B Cells in Cardiovascular Disease) network. Work in the laboratory of C. Kemper is supported, in part, by the Intramural Research Program of the National Institutes of Health (NIH), National Heart, Lung, and Blood Institute (ZIA/hl006223 to C. Kemper). Work in the laboratories of both C. Kemper and Z. Mallat is supported by the NIH-OXCAM (Oxford-Cambridge Scholars Program) Scholars Program and a Gates Cambridge Scholarship (A. Case). Z. Mallat and T.X. Zhao are supported by the British Heart Foundation (BHF), the BHF Centre of Research Excellence, and the NIHR (National Institute for Health and Care Research) Cambridge Biomedical Research Centre.

### Disclosures

None.
